# Monitoring ICU Mortality Risk with A Long Short-Term Memory Recurrent Neural Network

**Published:** 2020

**Authors:** Ke Yu, Mingda Zhang, Tianyi Cui, Milos Hauskrecht

**Affiliations:** 1 Department of Computer Science, University of Pittsburgh; 2 Intelligent Systems Program, University of Pittsburgh

**Keywords:** ICU Mortality Monitoring, Patient Representation, Latent Semantic Analysis, Long Short-Term Memory Recurrent Neural Network

## Abstract

In intensive care units (ICU), mortality prediction is a critical factor not only for effective medical intervention but also for allocation of clinical resources. Structured electronic health records (EHR) contain valuable information for assessing mortality risk in ICU patients, but current mortality prediction models usually require laborious human-engineered features. Furthermore, substantial missing data in EHR is a common problem for both the construction and implementation of a prediction model.

Inspired by language-related models, we design a new framework for dynamic monitoring of patients’ mortality risk. Our framework uses the bag-of-words representation for all relevant medical events based on most recent history as inputs. By design, it is robust to missing data in EHR and can be easily implemented as an instant scoring system to monitor the medical development of all ICU patients. Specifically, our model uses latent semantic analysis (LSA) to encode the patients’ states into low-dimensional embeddings, which are further fed to long short-term memory networks for mortality risk prediction. Our results show that the deep learning based framework performs better than the existing severity scoring system, SAPS-II. We observe that bidirectional long short-term memory demonstrates superior performance, probably due to the successful capture of both forward and backward temporal dependencies.

## 1. Introduction

Intensive care units (ICU) provide critical care and life support for most severely ill and injured patients in the hospital. Patients in ICU need to be monitored closely in order to detect the deterioration of patient’s condition or occurrence of various adverse events influencing the already fragile patient state. On the other hand, high demand for ICU services and limited bed availability have posted bed planning challenges. Physicians need to identify patients with the lowest risk for discharge to reduce ICU admission delays for new patients. With the availability of large healthcare databases, such as Medical Information Mart for Intensive Care (MIMIC-III), researchers have developed various scoring systems^1,2^ and machine learning models^3–5^ to assess patient mortality using a small set of hand-crafted features. More recently, deep learning models trained on structured clinical data have demonstrated promising mortality risk prediction performance.^6^ However, the majority of existing models do not account for dynamically changing patient condition and their prediction is a single score for the entire ICU stay, usually based on the observations within the first 24 or 48 hours after ICU admission.

To address the above challenges, we propose a new framework for dynamic monitoring of ICU patients’ mortality risk based on structured EHR data. Inspired by language-related models, we propose to use latent semantic analysis (LSA) based embedding to define the current state of the patient. The state is defined by a combination of physiological variables, laboratory tests and medications given over a period of time of fixed length. The intuition here is that the patient’s data that consists of sequences of events logged in time corresponding to various treatments, laboratory tests, medications, and vital signs, can be thought of as a document and the codes of these events can be thought of as words. We process the EHR data by retrieving the counts of all relevant medical events that occurred in each time block during the patient’s ICU stay and accumulate them using the bag-of-words (BoW) representation. Similarly to documents, the BoW representation lets us summarize the state of the patient in a specific block of time. In our work, we limit the sequence of past events to the most recent 48-hour period which is expected to be sufficient for assessment of medical condition of ICU patients with respect to patient mortality. We would also like to note that the BoW representation is robust and can cope with missing data that are very common in EHRs. Since the number of clinical events in the BoW representation can be enormous, we use (unsupervised) LSA methods to convert the BoW representation to a lower-dimensional embedding. In order to further compress the information useful for mortality prediction, we propose and explore various methods for summarizing sequences of patient states over the 48-hour history window. These include (1) average pooling, (2) self-attention mechanism, (3) hidden space of the long short-term memory networks (LSTM), (4) hidden space of bidirectional LSTM.

We experiment with our mortality monitoring framework and various history summarization methods on MIMIC III dataset. Our results show that, for the task of mortality monitoring, features extracted by our framework outperform the traditional features used in a severity scoring system, SAPS-II.^1^ Furthermore, we observe that summarization based on bidirectional LSTM yield superior results comparing to other history summarization approaches.

## 2. Background and Related Work

Two types of EHR data are used commonly to support various clinical predictions: unstructured (free text) data, and structured data recording complex sequences of various clinical events in EHRs. Unstructured data, such as, clinical notes contain summary of past or present patient’s condition, clinicians’ insights and interpretations of the patient case, as well as, treatment plans. Structured data record the detail of the patient case, that include sequences observations, measurements, findings, and other clinical events. Both structured and unstructured data (either individually or jointly) were successfully used to support a variety of prediction tasks such as patient mortality, length of stay, readmission prediction or coded diagnoses assignment. Examples of such work include Miotto et al^7^ work on the Deep patient model that uses structured and unstructured data to learn the low dimensional representation for length-of-stay, readmission and diagnoses predictions, Perrote et al^8^ for assignment of diagnoses based on text data, Malakouti and Hauskrecht^9^ for assignment of diagnoses and diagnostic categories based on structured EHR data, and many others. In this work, we study methods based on structured data and focus on the mortality prediction problem.

Mortality prediction problem was initially introduced to predict the final in-hospital mortality using early EHR data such as the first 24 hours after ICU admission. Some researches have demonstrated advanced results utilizing more flexible periods of data during the entire encounter. Johnson et al^10^ proposed a distinct sampling scheme to extract data from a random time window, which generated a model applicable to real-time mortality prediction on the eventual mortality risk. Ho et al^11^ developed an interpretable RNN method using Learned Binary Mask to dynamically predict ICU mortality risk at the end of ICU encounters. Departing from these studies, in this paper, the mortality monitoring task we propose operates continuously across the entire encounter instead of making one prediction per encounter.

The success of a predictive algorithm largely depends on the quality of features representing the data.^12^ EHR data are challenging to represent and model due to its high dimensionality, noise, incompleteness, and heterogeneity. Recent developments in deep learning models allow us to address some of these challenges and unlock the information in the EHR. Miotto et al^7^ used a three-layer stacks of denoising autoencoders to capture hierarchical regularities and dependencies in the aggregated EHRs. Rajkomar et al^13^ demonstrated that deep learning methods using patients’ entire raw EHRs are capable of accurately predicting multiple medical events from multiple centers without site-specific data harmonization. Lipton^14^ proposed to apply LSTM models on EHR data, empirically evaluating its capability in recognizing patterns in multivariate time series of clinical measurements. Choi^15^ built a GRU-RNN deep learning model to detect early heart failures with a relatively long observation window ranging from 12 to 19 months. Che^16^ proposed an interpretable mimic learning framework to predict mortality and ventilator-free days, which used the learned feature representation or soft labels obtained from the deep learning models (based on GRU and DNN) to train the gradient boosting tree based mimic model. Song^17^ proposed and studied a full attention-mechanism-based sequence modeling architecture for multivariate time-series data, SAnD, and showed it has similar effectiveness as LSTM-based model approaches.

## 3. Data and Preprocessing

### 3.1. Data Source and Cohort Selection

We use data from the Multiparameter Intelligent Monitoring in Intensive Care (MIMIC-III v.1.4), which is a publicly available dataset^18^ that includes all patients admitted to an ICU at the Beth Israel Deaconess Medical Center from 2001 to 2012. One part of the MIMIC-III dataset was extracted from the CareVue system which archived data of patients who were admitted in years 2001–2008, the other part was extracted from the MetaVision system and covers patients admitted to ICUs in years 2008–2012. To avoid mapping of the two disjoint coding systems, in this work, we chose only admissions recorded in the MetaVision system. This lead to 22,049 unique in-hospital admissions of which 10.5% ended-up dying and 89.5% were discharged after treatments.

### 3.2. Data Extraction and Preprocessing

We extract three different types of events: laboratory test, vital signs and medications from the following tables: LABEVENTS, CHARTEVENTS and INPUTEVENTS_MV. We derive the label of death event for a patient using the field DEATHTIME, which is only present if the patient died in hospital, from the table ADMISSIONS.

In the LABEVENTS table, FLAG indicates whether the laboratory value is considered as abnormal or not. We split abnormal cases into ‘abnormally high’ or ‘abnormally low’ using the mean of normal values as the threshold. When creating labels for laboratory test, we concatenate ITEMID and the derived FLAG from the LABEVENTS table, so that events with normal results, events with abnormally high and abnormally low results are explicitly distinguished. Similarly, when creating labels for vital signs, we add a special tag (append a letter “W” to ITEMID) to indicate whether the WARNING is labeled by a caregiver on patient’s chart. We only use ITEMID to represent all the medications and ignore the dosage and other information. For continuous infusion over a certain period of time, we count its ITEMID once each time block within that period. This data preprocessing yields 1,147 unique labels of laboratory test, 2,798 unique labels of vital signs and 277 unique labels of medications. The vocabulary size of all labels is 4,222.

## 4. Methodology

### 4.1. Mortality Monitoring Task

Our objective is to define a framework for monitoring mortality risk from past patient observations in EHRs. A formal definition of the problem is as follows: for a specific patient *p*, and a time series of past clinical events *e*^(*t*1)^*, e*^(*t*2)^*, …, e*^(*tn*)^ observed at times *t ∈ {t*_1_*, t*_2_*, …, t*_*n*_*}* for that patient prior to the current time *T*, predict whether a specific event of interest *d* is going to happen in the next prediction window of size *T*_*p*_, i.e., [*T, T* + *T*_*p*_]. Note that many different types of past clinical events can be considered, for example, administration of the different medications, observations of various laboratory test results or observed physiological signals.

To define our framework we consider a limited history of past events to predict the future event of interest. That is, at any given current time point *T*, our framework looks back in time for a fixed time span *T*_*h*_, i.e., [*T − T*_*h*_*, T*], and retrieves all the relevant medical events *{e*^(*t*)^*}* that occur during this time period, where *t ∈* [*T − T*_*h*_*, T*]. The prediction of the target event *d* then relies only on the events in this time window. As time progresses both history window defined by *T*_*h*_ and prediction window defined by *T*_*p*_ move according to the current time *T*, thus our framework always predicts the event in the near future.

Capturing the changes in patient’s condition is important for our task. To that end, we divide the history window [*T − T*_*h*_*, T*] into *k* equal-sized time blocks *b*^(1)^*, b*^(2)^*, …, b*^(*k*)^, so that trends or other dynamic patterns can be effectively modeled. For each *b*^(*j*)^, we use LSA^19^ projections to obtain low-dimensional representation $x^{\left(b_{j}\right)}$ of the events in block *b*^(*j*)^ from the counts of all medical events $\left\{e^{\left(b_{j}\right)}\right\}$ that occur during this time block. We apply LSA as an effective technique for representing complex patient’s state covering many different events. Specifically, we take the BoW representations from all available time blocks as inputs and then apply singular value decomposition (SVD) to perform dimensionality reduction. The obtained LSA embeddings are then standardized (zero-mean, unit variance).

Our next step is to further summarize information from the sequence of embeddings *x*^(1)^*, x*^(2)^*, …, x*^(*k*)^ reflecting the sequence of recent patient’s states occurring during the history window *T*_*h*_ into a vector representation *h*_*seq*_ that can accurately predict patient’s near term mortality risk. We explore various methods to combine and summarize the sequence data: (1) average pooling, (2) self-attention, (3) LSTM, (4) bidirectional LSTM. The details of these methods are provided in the following sub-sections.

### 4.2. Average Pooling and Attention Mechanism

Several common strategies for combining sequential data include average pooling, max-pooling or concatenation. However, all these methods treat data representing individual sequence steps equally when summarizing the sequence. Briefly, in our framework the sequence is defined by a sequence of *k* most recent patient states covering (equally) the history window of size *T*_*h*_. Hence, under the average pooling all these states would be treated equally and the final representation is defined as:

(1) $$h_{\text{seq}}^{\text{avg}}\;=\text{ average}\left(x^{\left(b_{1}\right)},x^{\left(b_{2}\right)},\ldots ,x^{\left(b_{k}\right)}\right)$$

To permit more flexible representation of a patient state sequence, we adopt the idea of self-attention^20^ that allows the model to automatically focus its attention on more interesting predictive patterns. More specifically, we create a stand-alone module with shared parameters to inspect individual representation vector, and the module outputs a score as an indication of the significance of that representation. The intuition comes from the observation that experienced medical specialists could easily find abnormal results from medical tests, pay attention to changes in time and focus on a specific time period based on the signals. The self-attention matrix *W*_*a*_ is trained end-to-end with the main prediction loss (mortality-based loss), and the normalized attention score is multiplied back to the representation. More specifically, for each representation $x^{\left(b_{j}\right)}$, the corresponding “attention” score $\alpha^{b_{j}}$ is calculated as:

(2) $$\alpha^{\left(b_{j}\right)}=\text{softmax}\left(W_{a}x^{\left(b_{j}\right)}\right),\;j\in [1,k]\;h_{\text{seq}}^{\text{attn}}=\overset{k}{\underset{j=1}{\sum }}\alpha^{\left(b_{j}\right)}x^{\left(b_{j}\right)}$$

Briefly, the final representation of the entire sequence, $h_{\text{seq}}^{\text{attn}}$, is a function of the individual sequence components $x^{\left(b_{j}\right)}$, weighted by the attention weights *α*.

### 4.3. Recurrent Neural Network (RNN)

Recurrent neural networks let us model and learn input-output sequences with the help of a hidden state. In this work, we consider LSTM^21^ to model RNN. LSTM has demonstrated superior performance for modeling sequential data due to the internal gating mechanism preventing the vanishing and exploding gradient calculations. By incorporating “input gate”, “output gate” and “forget gate”, LSTM cell learns to control the information flow, thus increasing its capability for handling long-term input-output dependencies. The specific gating operations are defined below. *i*, *f*, *o* represents “input”, “output” and “forget” gates respectively, and *W* and *U* are trainable parameter matrices. Note that ☉ denotes element-wise multiplication, and *σ* and tanh denote commonly used non-linear activation functions.

(3) $$i^{\left(b_{j}\right)}=\sigma \left(W^{\left(i\right)}x^{\left(b_{j}\right)}+U^{\left(i\right)}h^{\left(b_{j}-1\right)}\right)\;f^{\left(b_{j}\right)}=\sigma \left(W^{\left(f\right)}x^{\left(b_{j}\right)}+U^{\left(f\right)}h^{\left(b_{j}-1\right)}\right)\;o^{\left(b_{j}\right)}=\sigma \left(W^{\left(o\right)}x^{\left(b_{j}\right)}+U^{\left(o\right)}h^{\left(b_{j}-1\right)}\right)\;{\tilde{c}}^{\left(b_{j}\right)}=\text{tanh}\left(W^{\left(c\right)}x^{\left(b_{j}\right)}+U^{\left(c\right)}h^{\left(b_{j}-1\right)}\right)\;c^{\left(b_{j}\right)}=f^{\left(b_{j}\right)}⊙{\tilde{c}}^{\left(b_{j}-1\right)}+i^{\left(b_{j}\right)}⊙{\tilde{c}}^{\left(b_{j}\right)}\;h^{\left(b_{j}\right)}=o^{\left(b_{j}\right)}⊙\text{tanh}\left(c^{\left(b_{j}\right)}\right)\;h_{\text{seq}}^{\text{lstm}}=h^{\left(b_{k}\right)}$$

In our work, we use LSTM and its hidden state to summarize the sequences of patient states. Briefly, after obtaining the LSA embeddings for each block in the recent history window, the embeddings are sequentially fed to the LSTM model step by step following their temporal order. The hidden state generated by the LSTM for the last block $h^{\left(b_{k}\right)}$ is then used as a representation of the entire embedding sequence $h_{\text{seq}}^{\text{lstm}}$ and supports patient mortality monitoring.

Inspired by the recent advancements in natural language processing,^22–24^ we also explore bidirectional RNNs.^25^ The bidirectional RNNs were introduced to better handle long-term dependencies in sequences. Briefly, even with the help of LSTMs, the RNN models may forget some of the early signals in the sequence. A trick to fix this problem is to build two RNNs, one digesting the sequence forward following the temporal order, and the other one backwards. The final sequence representation is then obtained by concatenation of the forward and backward hidden states.

## 5. Experimental Design

### 5.1. Sampling Strategy

**Fig. 1.**
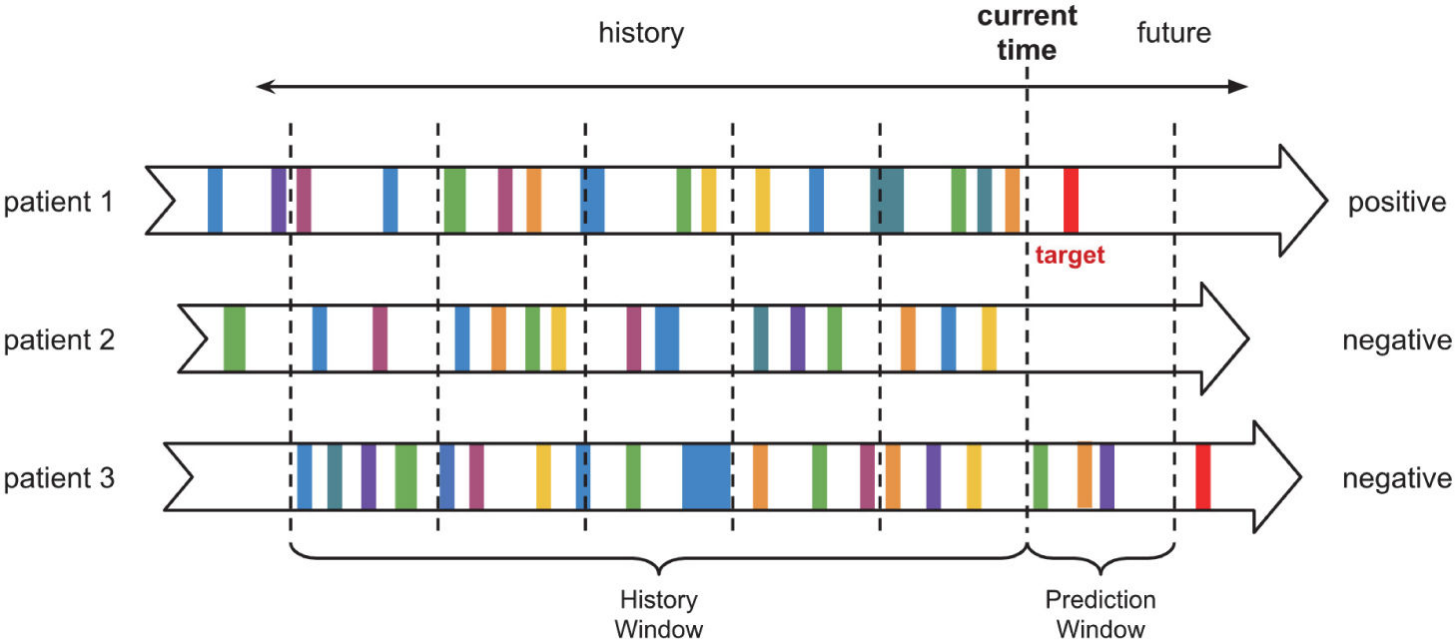
Illustration of a strategy for extracting positive and negative instances from admission records. Bands with different colors represent different medical events (with varied duration), and the whole record is split by predefined block size (dotted line). At any given time, the most recent events within the history window are used to define features and the occurrence of the target event (death) in the prediction window defines the label. Notice, that the instance that is generated for Patient 3 is labeled negative, although the patient eventually dies. This is because the future event is assessed purely by the prediction window.

After data preprocessing, as described in Section 3.2, we extract actual training samples from admission records. Since our goal is to assess the mortality risk of ICU patients continuously, we divide each admission record into multiple history windows, each of which can be seen as a single training instance. The label for each instance depends on whether the target event (death) appears in the corresponding prediction window. In this way, the original dataset that consists of admissions is converted into individual instances, as illustrated in Figure 1. Please note that we do not generate any instance for the patient whose admission record is shorter than the history window.

Since multiple instances can be sampled by moving the history window over a single admission, the number of total negative instances is much higher than the number of positive instances that only occur if patients died at the end of ICU stay. To deal with unbalanced data and learn features predictive of mortality, we keep all positive instances and down-sample an equal number of negative instances while training the final logistic regression models. However, the results on the test set are always based on all instances generated for the testing admissions.

### 5.2. Baseline Model

SAPS-II^1^ score is designed to measure the severity of the disease for ICU patients using data collected within the first 24 hours of admission. From the prediction window of each instance, we extract and process features used in the calculation of the SAPS-II score, including serum urea nitrogen level, white blood cells count, serum bicarbonate level, sodium level, potassium level, urine output, pao2/fio2 ratio, body temperature, heart rate, systolic blood pressure, Glasgow coma scale, indicators of chronic diseases and admission type. We perform mean imputation to fill-in missing values. We exclude the bilirubin level score, since its missing rate is too high. We fit a logistic regression model using the SAPS-II features as the baseline for comparison with other models using data-driven feature representations.

### 5.3. Experimental Settings

We first randomly divide the MIMIC dataset according to the admission ID into three disjoint subsets representing train, validation and test sets. This yields 13,344 admissions used for training, 4,329 admissions for validation and 4,376 admissions for testing. We use the block size of 6 hours (*T*_*b*_ = 6), as picking a too small block size leads to very sparse bag-of-words representations while using an excessively long block size diminishes important trend signals. We set the length of history window to 48 hours or 8 blocks (*k* = 8, *T*_*h*_ = 48) based on a comparison analysis, which shows history window of 48 hours slightly outperforms history window of 24 hours. We set the length of the prediction window to 12 hours or 2 blocks (*T*_*p*_ = 12). The methods in the paper are implemented using PyTorch,^26^ and we use RMSProp^27^ for optimization with the initial learning rate set to 0.001. We use a single hidden layer of size 32 in both LSTM and bidirectional LSTM experiments.

For feature representation, we experiment with different architectures from simple average pooling to more complicated recurrent neural network. For a fair comparison, all methods use a logistic regression model for binary classification. For evaluation metrics, we use the area under the receiver operating characteristics (ROC-AUC score) and Precision-Recall Curve (or Average Precision) to report the model’s performance. The results are reported on all instances generated for test admissions that include 19,107 negative instances and 634 positive instances.

## 6. Results and Analysis

### 6.1. Dimensionality Analysis

We experimented with the dimensionality of the LSA embeddings on the training and validation data. We found that the embedding size of 256 yields the best performance using the LSTM as the method for feature representation. Our experiments show that over-reduced dimensionality on the input embeddings hurts prediction performance, while over-supplying redundant information (e.g., LSA embedding size of 512) would likely cause overfitting.

We also experimented with the different hidden state-space size. We observed that LSTM is able to find a more compact sequence representation and achieves the best performance with the hidden state-space size of 32. Based on these experiments (based on training and validation data), we fixed the LSA embedding size at 256 and the LSTM hidden state size at 32.

### 6.2. Prediction with Different Feature Representations

Table 1 shows the in-hospital mortality prediction results for the different feature representation methods. We observed that: (1) data-driven feature representations consistently outperform SAPS-II features, (2) self-attention mechanism slightly outperforms average pooling by using weighted sum of individual representations, (3) the recurrent neural networks obtain better results by learning the temporal dependency in the sequence data, in which the bidirectional LSTM achieves the best AUROC and AUPRC scores. Note that the results presented in Table 1 are not strictly comparable with the benchmarking results reported by other modern mortality prediction models,^4,6^ because their task is to predict the patient’s mortality risk at the end of the ICU stay while ours is to monitor the mortality risk of the patient continuously.

**Fig. 2.**
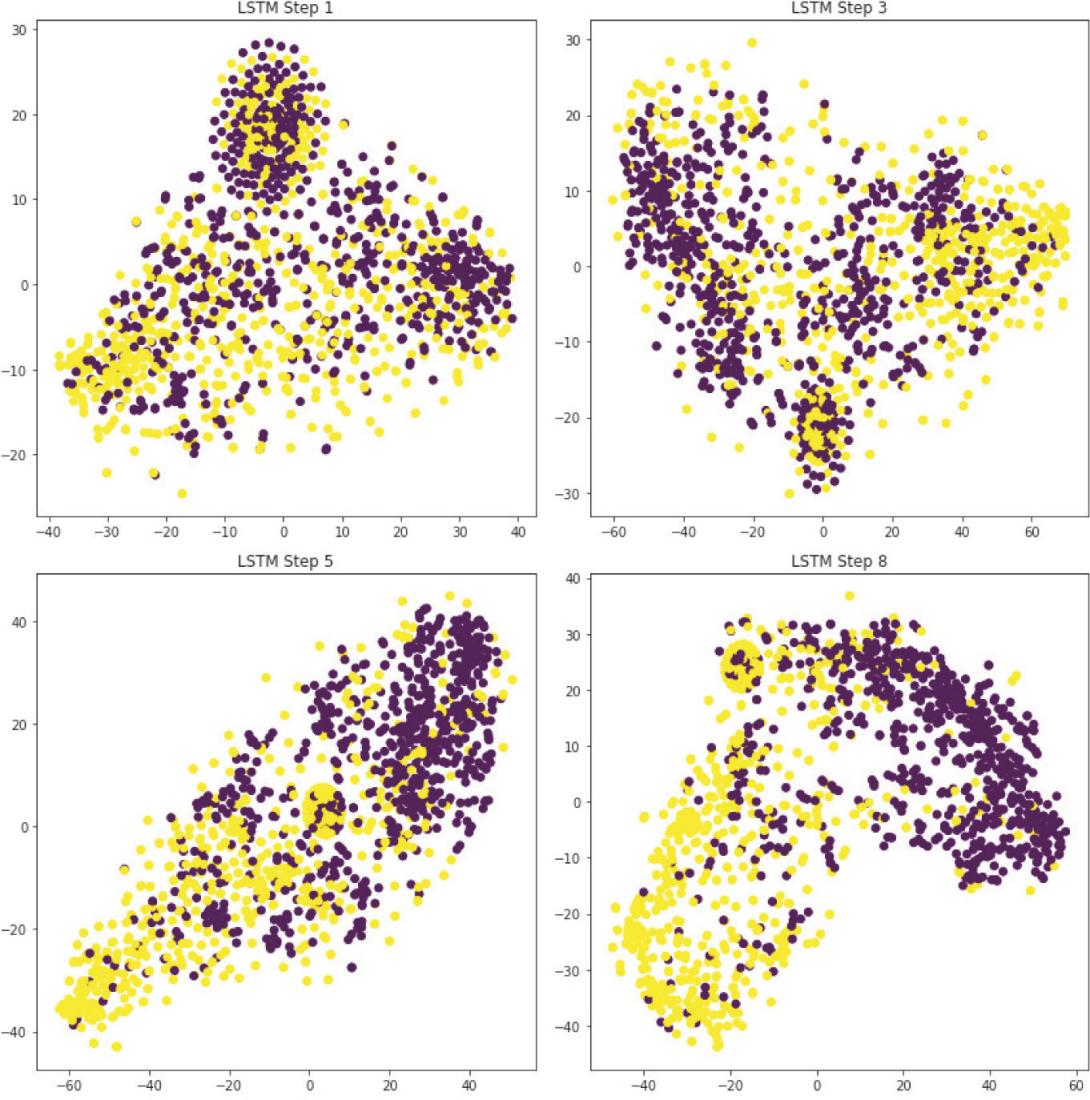
Visualization of hidden states at various time steps in unidirectional LSTM using two dimensional t-SNE. Yellow dots represent the instances of positive class (death) and purple dots represent the instances of negative class. Note that the classes are progressively separated out over time steps in unidirectional LSTM.

**Fig. 3.**
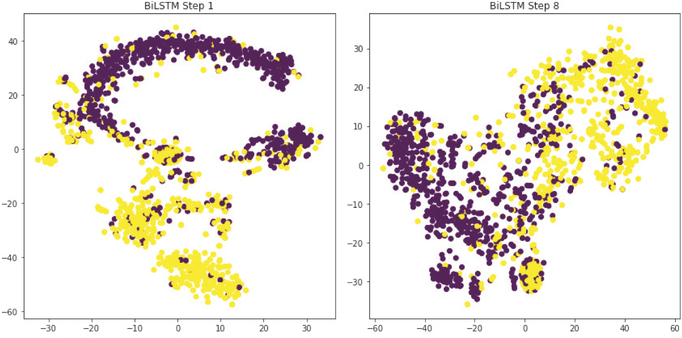
Visualization of first and last hidden states in bidirectional LSTM using two dimensional t-SNE. Yellow dots represent the instances of positive class (death) and purple dots represent the instances of negative class. Representations learned from forward module (right figure) and backward module (left figure) of bidirectional LSTM exhibit different structures in the reduced 2D space. Both of them are useful for separating the positive and negative instances.

We used t-Distributed Stochastic Neighbor Embedding^28^ (t-SNE) as a visualization tool to project the hidden states $h^{\left(b_{j}\right)}$ of the LSTM models into lower dimensions. t-SNE is a non-linear dimensionality reduction technique, which captures local structures of the original high-dimensional data while revealing global structures in the meantime. The shorter distance of two points in the resulting low dimensional figure represents the higher similarity of the original data. Figure 2 demonstrates that unidirectional LSTM works dynamically and is able to discover increasingly more effective representations over time steps. From Figure 3 we observed that: (1) the forward module of bidirectional LSTM learned almost the same structure as the unidirectional LSTM at the last time step (with 180° rotation); (2) the representation learned by the backward module of bidirectional LSTM exhibits a different structure than its forward representation, and may help the model achieve superior results; (3) Both the forward and backward module learn linearly-separable patterns from the LSA embeddings.

### 6.3. Interpreting Mortality of Learned Representation

**Fig. 4.**
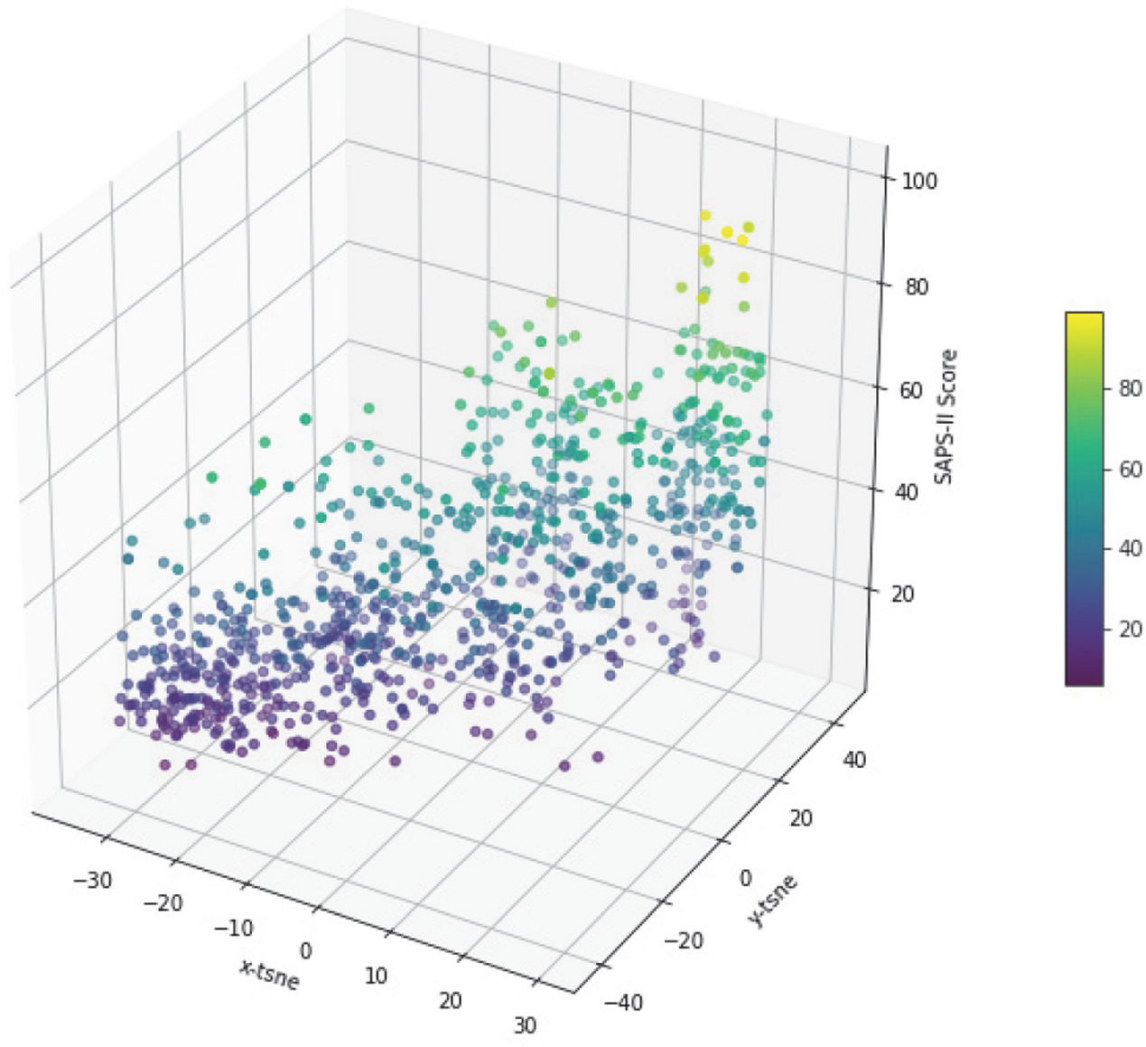
Relationship between SAPS-II score and bidirectional LSTM hidden states transformed by t-SNE. X, Y axis are the two embeddings selected from the t-SNE transformation. Z axis as well as the color bar show the value of SAPS-II score. The hidden states generated by bidirectional LSTM are correated with SAPS-II scores.

We also analyzed how the feature representations learned from the RNN models relate to medical significance. In Figure 4 we show an interesting pattern that SAPS-II scores, which are the sum of all the SAPS-II features used in the baseline model, are highly correlated with the sequence representation $h_{\text{seq}}^{\text{lstm}}$ of our bidirectional LSTM at the last time step. Specifically, in the xy-plane, we plot the learned representations transformed by t-SNE, and the z-axis represents the SAPS-II scores of that particular instance. The hidden states generated by our bidirectional LSTM are correlated with SAPS-II scores; patients with high scores are located in the upper-right region of the hidden space, and patients with low scores are more concentrated in the lower-left region. Note that the sequence representation generated by the bidirectional LSTM only uses the labels of medical events as source inputs, and is never exposed to the explicit values used in the calculation of SPAS-II score, such as heart rate, urine output, WBC count and etc. Nevertheless, our approach is able to capture clinically meaningful representations in a latent space generated by our recurrent neural network.

## 7. Discussion and Conclusions

This paper proposes an approach to closely monitor patients’ mortality risk using the most recent medical history of structured EHR data. The new framework can be used as an instant scoring system that helps ICU clinicians assess the severity of illness of patients in ICUs. Inspired by language-related models, we use counts of all relevant medical events as source features that are robust to missing data, and apply latent semantic analysis (LSA) to obtain a compact embeddings to represent patients states. To better model the time dependencies within a specific history window, we experimented with various feature representation methods, including average pooling, self-attention mechanism and hidden states of various LSTM models. Notably bidirectional LSTM demonstrates the most competitive performance, partly because it explicitly takes both forward and backward temporal information into consideration. Our exploration shows that using data-driven approach with very little human intervention is able to achieve accurate predictions. We expect our method to generalize well also for prediction of other critical events (e.g. sepsis) and we plan to investigate these in the future.

Although promising, our approach also comes with several limitations. First, medical events we used to construct feature representations were discrete-valued. By considering exact numerical information associated with medical events, such as lab test results, vital sign readings, or dosage information our models may further improve. Second, we did not use (free-text) progress notes, which contain rich information about the patient and are also less prone to coding errors. Finally, in the medical domain it is important to consider the interpretability of feature representations. Whilst hidden states of LSTM models are effective for risk stratification, they are also difficult to trace back to the original medical events.

## Figures and Tables

**Table 1.** Prediction performance for the different feature representation strategies. Binary classification is performed by a logistic regression. The best performing models are highlighted in **bold**.

Model	AUROC	AUPRC
SAPS-II	0.7749	0.1051
Average Pooling	0.8359	0.2612
Self-Attention	0.8360	0.2679
Unidirectional LSTM	0.8783	0.3092
Bidirectional LSTM	**0.8854**	**0.3184**
